# A Reward-Based Framework of Perceived Control

**DOI:** 10.3389/fnins.2019.00065

**Published:** 2019-02-12

**Authors:** Verena Ly, Kainan S. Wang, Jamil Bhanji, Mauricio R. Delgado

**Affiliations:** ^1^Institute of Psychology, Leiden University, Leiden, Netherlands; ^2^Leiden Institute for Brain and Cognition, Leiden, Netherlands; ^3^Department of Psychology, Rutgers University – Newark, Newark, NJ, United States; ^4^Behavioral and Neural Sciences Graduate Program, Rutgers University – Newark, Newark, NJ, United States

**Keywords:** perceived control, controllability, choice, instrumental behavior, reward rate, corticostriatal circuit, striatum, dopamine

## Abstract

Perceived control can be broadly defined as the belief in one’s ability to exert control over situations or events. It has long been known that perceived control is a major contributor toward mental and physical health as well as a strong predictor of achievements in life. However, one issue that limits a mechanistic understanding of perceived control is the heterogeneity of how the term is defined in models in psychology and neuroscience, and used in experimental settings across a wide spectrum of studies. Here, we propose a framework for studying perceived control by integrating the ideas from traditionally separate work on perceived control. Specifically, we discuss key properties of perceived control from a reward-based framework, including choice opportunity, instrumental contingency, and success/reward rate. We argue that these separate reward-related processes are integral to fostering an enhanced perception of control and influencing an individual’s behavior and well-being. We draw on select studies to elucidate how these reward-related elements are implicated separately and collectively in the investigation of perceived control. We highlight the role of dopamine within corticostriatal pathways shared by reward-related processes and perceived control. Finally, through the lens of this reward-based framework of perceived control, we consider the implications of perceived control in clinical deficits and how these insights could help us better understand psychopathology and treatment options.

## Introduction

Decades of research focusing on the perception of control have highlighted its importance in general well-being, in particular being predictive of life achievements and health (Skinner, 2007). For instance, perceived control is associated with better career prospects and job performance (Stillman et al., 2010), while disruptions in perceived control constitute a core characteristic of many psychiatric disorders, such as anxiety and depression (Gallagher et al., 2014a; Liu et al., 2016). One issue that limits a mechanistic understanding of perceived control and how it positively benefits behavioral and health outcomes is the heterogeneity of how the term is used and applied in experimental settings across a wide spectrum of studies (e.g., see Table 1). Several theories have been posited to explain the construct of perceived control (for review see Skinner et al., 1996). For instance, the term “locus of control” was coined to describe differences in individual beliefs that situations are either within (i.e., internal locus of control) or outside one’s control (i.e., external locus of control; Rotter, 1966). Along a similar vein, the concept of self-efficacy was introduced to capture the belief in one’s ability to exercise control over the external environment (Bandura, 1977). Collectively, these theories on perceived control can be interpreted to refer to the same underlying phenomenon: perceived control is the belief in one’s ability to exert control over situations or events in order to gain rewards and avoid punishments.

**Table 1:** Selection of perceived control-related terms.

Term	Definition	Relevant literature
Locus of control	Belief that situations are within (internal) or outside (external) one’s control	Rotter, 1966
Self-efficacy	Belief in one’s ability to exercise control over the external environment	Bandura, 1977
Self-determination	Experience of choice; be the determinants of one’s actions	Deci and Ryan, 1985
Effectance motivation	Fundamental drive to have an influence on our environment through our own actions	White, 1959

This interpretation of perceived control is closely related to the definition of instrumental contingency based on reinforcement learning principles, where behavioral strategies and actions are reinforced by contingent desired outcomes (Thorndike, 1911; Shanks and Dickinson, 1991; Dickinson and Balleine, 1994; Liljeholm et al., 2012). In line with this interpretation, contemporary accounts based on learning theories propose that the perception of control is formed through learned relationships between actions and contingent outcomes, as well as the generalization of inferred controllability from these relationships to novel situations (Huys and Dayan, 2009; Lieder et al., 2013; Rigoli et al., 2016; Moscarello and Hartley, 2017). Thus, these accounts emphasize the role of inference of controllability in the development of perceived control.

However, there are some challenges to the notion that instrumental action-outcome contingency is the sole driver of perceived control. For instance, it has been suggested that aspects related to the outcome itself, such as the average reward or its frequency, may also play a role in perceived control (Alloy and Abramson, 1979; Teodorescu and Erev, 2014), and that the mere opportunity for control is desirable and linked to reward-related neural systems (Fujiwara et al., 2013; Leotti and Delgado, 2011, 2014). This work converges with psychological theories suggesting that perceived control reflects a fundamental psychological need and desire for control (White, 1959; Deci and Ryan, 1985; Elliot and Dweck, 2005) and highlights the importance of affective and motivational properties in understanding the perception of control. More specifically, rather than solely focusing on the role of instrumental action-outcome contingencies, this reward-based framework emphasizes that the drive to exercise control through choice is key to fostering perceived control. It is consistent with observations that the belief or perception of control is more powerful in predicting decision making and behavioral consequences than having objective control (i.e., the *actual* existence of action-outcome contingencies; Averill, 1973; Abramson et al., 1978; Skinner et al., 1996; Eitam et al., 2013). Even with the absence of objective control, having the perception of control is sufficient to increase arousal and mobilize action; whereas perceiving the lack or loss of control leads to helplessness despite the presence of objective control (Averill, 1973; Abramson et al., 1978; Skinner et al., 1996). Taken together, the literature suggests that the affective and motivational properties of choice and outcome may play an important role in the development of perceived control via their impact on instrumental decisions and the inference of control.

**FIGURE 1:**
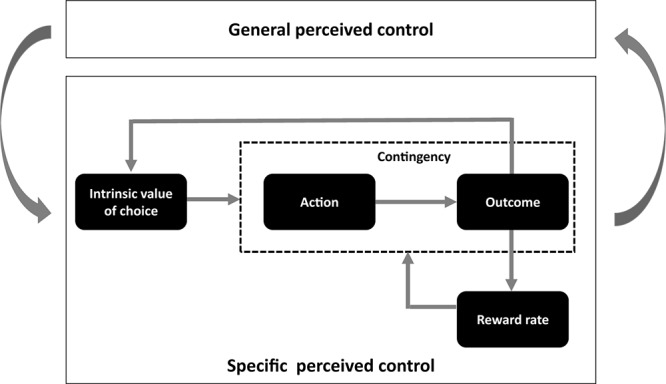
Key elements contributing to context-specific and general perceived control. An inherent appetitive value of choice could support a natural drive to seek out choices and situations that confers actual control. Learning-related prediction error signals accompanying desired outcomes following our actions can reinforce our preference for choice. Following from these processes, an increase in reward rate could further contribute to learning by enhancing alertness and motivational drive. In a loop, these processes that take place in specific contexts could influence more abstract general beliefs about perceived control via neuroplasticity mechanisms within the corticostriatal pathways and transient changes in baseline levels of dopaminergic transmission.

In the current review, we attempt to present a more inclusive reward-based framework of perceived control by integrating the ideas from the traditionally separate work on perceived control. We argue that distinct reward-related processes, including choice opportunity, instrumental contingency, and success/reward rate, are integral to the fostering of a perception of control that influences behavior and well-being (Figure 1). We highlight how these reward-related factors are central to many investigations into perceived control. Then, we deliberate how the aforementioned reward-related elements are implicated separately and collectively in our understanding of perceived control. We particularly emphasize the overlapping role of dopamine within corticostriatal pathways in both reward processing and perceived control. Finally, through the lens of this reward-based framework of perceived control, we consider the implications of perceived control in clinical deficits.

## Choice Opportunity and Its Affective and Motivational Properties

The impact of perceived control on adaptive behavior and mental well-being has been suggested to reflect a basic psychological need for control (White, 1959). There exists a fundamental drive to influence on our environment via our own actions, so-called effectance motivation, that enables us to effectively interact with our world (White, 1959; Elliot and Dweck, 2005). For example, animals and humans show a clear preference for choice over no-choice situations, even when the choice option requires greater effort expenditure and does not increase outcome value (Catania and Sagvolden, 1980; Suzuki, 1997, 1999; Bown et al., 2003; Leotti et al., 2010). These behavioral studies are reinforced by neural data supporting the idea that choice opportunity carries an inherent appetitive value. For example, human participants showed greater activation in corticostriatal regions in response to the anticipation of choice compared to no-choice cues (Leotti and Delgado, 2011; Leotti and Delgado, 2014). This striatal activation has been further shown to track the increasing value of the opportunity to choose (Fujiwara et al., 2013). Taken together, these studies showing that choice opportunity recruits reward-processing regions suggest that perceived control might have affective and motivational properties that make it valuable in and of itself.

The affective property of choice opportunity could play an important role in promoting exertion of autonomy. It has been suggested that a preference for exercising choice could support a natural drive to seek out choices and situations that allow actual control, and therewith foster the perception of control (Leotti et al., 2010). This notion is in line with theories on action-valence coupling (Panksepp, 1998; Boureau and Dayan, 2011; Cools et al., 2011). According to these theories, appetitive value supports the attainment of rewards by eliciting appetitively motivated behaviors (e.g., approach, engagement, active exploration), partly mediated by dopamine projections to striatal regions, particularly the nucleus accumbens (NAcc). Given this strong action-valence coupling, choice opportunity could elicit appetitively motivated behaviors via its inherent appetitive value. One implication of this idea is that, given the reflexive nature of the action-valence coupling, incidental cues associated with choice opportunity could bias our behavior more generally, akin to forms of Pavlovian-instrumental transfer involving corticostriatal regions (Corbit and Balleine, 2011; Griffiths et al., 2014). This is an intriguing hypothesis given the increasing work on motivational biases in decision making (Huys et al., 2011; Ly et al., 2013; Swart et al., 2017; Piray et al., 2018). Future work should focus on how choice opportunity can by itself bias behavior and cognition. Indeed, it has been shown previously that choice opportunity can influence more cognitive processes such as the recall of declarative information (Murty et al., 2015).

Although we propose that choice opportunity is intuitively associated with appetitive behaviors, the motivational properties of choice should not only be limited to the appetitive context but could be extended into aversive contexts as well. Striatal dopamine has been shown to be involved in aversively-motivated behavior (Faure et al., 2009). When rodents were subjected to controllable shocks, they show control-seeking behaviors such as exerting effort to escape, which were accompanied by elevated dopamine levels in the NAcc (Cabib and Puglisi-Allegra, 2012). One potential suggestion raised by these findings is that even when the overall context is aversive, the affective and motivational properties of choice opportunity could be beneficial under certain circumstances (e.g., when the aversive outcome is controllable, such as in an avoidance task). Consistent with these animal studies, using a choice preference task in the context of potential monetary loss only, individuals demonstrate a preference for choice over no choice accompanied by a relative increase in striatal activation during choice anticipation (Leotti and Delgado, 2014). Thus, similar to choice preference in an appetitive context, individuals prefer choice over no choice in order to have control over aversive outcomes.

Moreover, there is evidence that choice opportunity could support the regulation of affect and behavior in an aversive context. For instance, individuals have been shown to be more effective at reducing picture-induced negative affect when they make a free choice, compared to when they are instructed to regulate their affect (Kühn et al., 2014). Additionally, it has been shown that free choice versus forced choice enhances performance, even if the choice element is task-irrelevant (Murayama et al., 2013). This performance enhancement in the choice condition was related to increased ventromedial prefrontal activation associated with resilience to failure feedback during the choice condition. Taken together, these findings demonstrate that choice opportunity could play an important role in promoting perceived control in both appetitive and aversive contexts.

## Instrumental Contingency Shapes Controllability

Although perceived control can have more impact on behavioral outcome than objective control, the existence and experience of actual control could constitute another element shaping our perception of control. In this section, we highlight how (past) experience of actual control could shape both context-specific and general perceived control using literature based on reinforcement learning principles as well as the learned helplessness model.

### Reinforcement Learning and Context-Specific Controllability

Objective or actual control is closely related to the definition of instrumental contingency based on reinforcement learning principles, where behavioral strategies and actions are reinforced by contingent desired outcomes (Thorndike, 1911; Shanks and Dickinson, 1991; Dickinson and Balleine, 1994; Liljeholm et al., 2012). These types of behaviors may allow us to flexibly regulate our environment and is critical to adaptive decision making. Following from this, perceived control in a specific context may arise when specific actions lead more deterministically and reliably to desirable and specific outcomes (Huys and Dayan, 2009; Lieder et al., 2013; Rigoli et al., 2016). Learning about these instrumental action-outcome contingencies have been linked to corticostriatal circuits and dopaminergic prediction error signals (Balleine and Dickinson, 1998; Hollerman and Schultz, 1998; Corbit and Balleine, 2003; Yin et al., 2005; Berridge, 2007; Valentin et al., 2007; Tanaka et al., 2008; Liljeholm et al., 2011; Griffiths et al., 2014). Thus, reinforcement learning involving corticostriatal circuits could provide the neurobiological basis of context-specific perceived control.

Interestingly, reinforcement learning and choice opportunity could also interact. By leveraging knowledge from a biophysical model of the striatum and reinforcement learning modeling, it has been shown that choice opportunity (versus no choice) is associated with increased dopamine-mediated positive prediction errors (Cockburn et al., 2014). These findings suggest that dopamine signals targeting areas in the striatum may be important for reinforcing the preference for control. Additionally, a recent study demonstrated that a preference for free choice was more pronounced in case of high instrumental divergence (i.e., when choices differ more with respect to their outcome probability distributions) (Mistry and Liljeholm, 2016). Thus, the preference for free choice was higher when choices have a more meaningful impact on outcome. These findings highlight that choice opportunity and instrumental reinforcement processes collectively contribute to perceived control.

### Generalization Processes of Controllability

Repeated experience of context-specific controllability could shape an abstract general (context-independent) belief about controllability, which in turn can guide our decisions and affect contingency learning in new situations, thus further contributing to our perception of control (Huys and Dayan, 2009; Lieder et al., 2013; Huys et al., 2015; Rigoli et al., 2016). In other words, prior experience with controllability could shape our current perception of control. Indeed, young developing nonhuman primates with control over receiving appetitive stimuli show enhanced active coping in later stressful situations, whereas those who received these appetitive stimuli noncontingently show increased anxiety and reduced exploratory behavior in novel situations (Goodkin, 1976; Mineka et al., 1986). These findings suggest that controllability over appetitive outcomes is associated with more future perceived control and protective effects, whereas uncontrollability over appetitive outcomes is associated with reduced perceived control in new contexts. Moreover, these findings support the idea that diminished perceived control may become relatively crystallized over time leading to psychopathology (Mineka and Hendersen, 1985; Chorpita and Barlow, 1998).

The process of generalization could play an important role in perceived control concepts such as a generalized loss of control in learned helplessness (Huys and Dayan, 2009). In the learned helplessness model, animals fail to escape from aversive stimuli in a novel context after being exposed to uncontrollable stressors, thus demonstrating a generalized loss of control (Miller and Seligman, 1975; Maier and Seligman, 2016). Importantly, the animals that experienced controllable stressors typically demonstrate a reduced stress response to immediate and subsequent external stressors. These protective effects of controllable outcomes have been shown to be mediated by projections from regions in the vmPFC (Vertes, 2004) to the dorsal raphe nucleus (DRN; Amat et al., 2005, 2006). Specifically, serotonergic activation in the DRN and its projections are involved in the expression of the behavioral response to stressors irrespective of its controllability (Maier and Watkins, 2005; Christianson and Greenwood, 2014). The controllability of the stressor is detected by the vmPFC, which plays a critical role in behavioral control over stressors by active inhibition of the DRN (Amat et al., 2005, 2006). It has been shown that experience of controllable stressors could increase excitability and plasticity proteins in the vmPFC that support the long-term increases in connectivity in behavioral control pathways (Varela et al., 2012; Christianson and Greenwood, 2014). These findings are supported by work in humans using a comparable paradigm: humans who experience escapable shocks, compared to inescapable shocks at the same rate, are quicker to extinguish conditioned responses to a stimulus that no longer predicts painful shock, and more likely to maintain extinction without spontaneous recovery on a later day (Hartley et al., 2014). Thus, experience of control over negative outcomes can have lasting effects on the way individuals learn from new experiences.

It has been suggested that the role of the vmPFC in mediating behavioral control over (future) stressors, that is to subserve the detection and perception of controllability, might be similar to its role in instrumental learning (Maier and Seligman, 2016). One study attempted to connect the separate lines of research on behavioral control and instrumental learning by demonstrating that the dorsomedial striatum, which is critically involved in instrumental learning, is required for behavioral control over stressors as well (Amat et al., 2014). Animal-to-human translational experimental studies focusing on the neural mechanisms underlying perceived control demonstrated that in humans too, the corticostriatal circuit, including the vmPFC and the striatum, were recruited during behavioral control over aversive stimuli (Salomons et al., 2004; Delgado et al., 2009; Kerr et al., 2012; Boeke et al., 2017). These findings support the idea that interactions between the vmPFC and the dorsomedial striatum play an important role in the detection of control, and subsequent vmPFC top-down regulation of the DRN allow for behavioral control over stressors (Maier and Seligman, 2016).

## Reward Rate, Tonic Dopamine, and (General) Perceived Control

The notion that instrumental action-outcome contingency is a key element of perceived control has been challenged by previous work (Alloy and Abramson, 1979; Teodorescu and Erev, 2014). Rather than learning about instrumental contingency *per se*, aspects related to the outcome itself, such as its size and frequency, may also play a role in perceived control. According to outcome-based accounts of perceived control, individuals regulate affect or behavior irrespective of knowledge about action-outcome contingencies when there is enough reward in our environment (Teodorescu and Erev, 2014). Conversely, individuals avoid the regulation of affect or behavior when reward is on average low. In other words, we tend to employ effortful regulation if reward rate is high, but we avoid such orientation altogether if reward rate is low (although see Niv et al., 2007, for interpreting the average reward rate more as an estimate of opportunity cost of time). Here, we discuss this alternative role of outcome-related aspects in perceived control.

The average reward rate and the associated changes in tonic dopamine levels could potentially contribute to the generalization process of controllability. Tonic dopamine has been suggested to reflect an average reward signal computed by slow averaging phasic prediction error signals (Niv et al., 2007): cues that have been associated with higher reward expectancies induce larger phasic positive prediction errors resulting in transient increases in tonic dopamine (Phillips and Wightman, 2004; Tobler et al., 2005). Although it has often been suggested that tonic and phasic dopamine are mediated by distinct mechanisms (Floresco et al., 2003; Bromberg-Martin et al., 2010), recent data demonstrated that phasic dopamine could trigger secondary events that increase tonic activation of dopamine levels (Lohani et al., 2018). It is therefore tempting to think that phasic dopamine signaling associated with higher reward expectancies or action values in context-specific perceived control, could influence general perceived control reflected by changes in tonic dopamine levels. In turn, transient changes in tonic dopamine levels could contribute to learning given its association with alertness and motivational drive (Schultz et al., 1997; Niv et al., 2007). These mechanisms may help to explain the interactions between general and context-specific perceived control. In line with these hypotheses, it has been suggested that tonic dopamine relates to trait-perceived internal locus of control (De Brabander and Declerck, 2004; Declerck et al., 2006; Kayser et al., 2014). Pharmacologically augmenting tonic dopamine restores exploratory behavior in individuals with an external locus of control characterized by reduced tonic dopamine (Kayser et al., 2014). Moreover, elevated levels of tonic dopamine have been observed in rodents in the context of controllable aversive outcomes, whereas prolonged exposure to uncontrollable aversive outcomes reduce tonic dopamine (Cabib and Puglisi-Allegra, 2012).

The suggestion that average reward rate relates to general perceived control resembles notions of effort-based decision making. Dopamine within the striatum is needed to sustain effort in order obtain desired outcomes (Niv et al., 2007; Beierholm et al., 2013). For instance, the administration of dopamine antagonist in the striatum of rodents decreases high effort responses for large rewards, but increases low effort responses for small rewards (Salamone, 2018). Computational accounts have explained these effects by relating the role of tonic dopamine levels to vigor versus sloth behavior and cost-benefit analyses (Niv et al., 2007; Phillips et al., 2007). Although we will not go further into the literature of effort-based decision making (for review, see Kurniawan et al., 2011; Salamone, 2018), this area of research is closely linked to aspects of general perceived control as presented here; both general perception of control and high effort could share similar underpinnings in tonic dopamine levels and motivational drive.

Interestingly, an alternative outcome-based account of perceived control that has been recently proposed suggests that reward prevalence (or frequency of outcome), rather than average reward rate, could explain exploratory behavior and perceived control (Teodorescu and Erev, 2014). Regardless of whether average reward rate or reward prevalence might be a better account for perceived control, outcome-based aspects could constitute an important element contributing to perceived control. One potential caveat of outcome-related processes in perceived control is revealed via contingency judgment tasks where the probabilities of an outcome and the probability of responding may create an ‘illusion of control’ (Orgaz et al., 2013; Tobias-webb et al., 2017). Consistent with the idea that a belief or perception of control is more potent than objective control, an illusion of control basically reflects the subjective judgment that an action-outcome causal relation exists when in fact there is no contingency. When probabilities of reward and action are high, the probability that both coincide is also high, hence affecting estimations of action-outcome causal relationships, which could contribute to the false belief that one has control (Alloy and Abramson, 1979; Matute, 1996; Orgaz et al., 2013).

## Clinical Implications

Perceived control deficit is a core feature in a number of psychiatric disorders. It has been suggested that disturbed perception of control contributes to a psychopathological state in a downward spiral, involving a dangerous cycle of poor decision making and stress exacerbation (Joiner et al., 2005). A reward-based framework of perceived control might shed light on the prevalence of perceived control deficits observed across clinical populations. In line with the aims of the Research Domain Criteria (Cuthbert and Insel, 2013), this framework may allow for the comparison of disorders marked by dysfunctions in basic reward-related processes using a transdiagnostic approach to better appreciate the underlying mechanisms of the behavioral problems associated with the lack or loss of perceived control. Based on the reward-based framework proposed in this paper, we suggest that perceived control deficits across disorders can be explained by dysfunctions in reward-related processing, which commonly implicate corticostriatal circuits and dopaminergic transmission. Below, we illustrate how disruptions in the key elements of the reward-based framework might explain the aberrant manifestations of perceived control in psychopathology.

### Disorders Characterized by Reduced Perceived Control

Arguably one of the most prevalent disorder to be associated with perceived control is major depressive disorder where patients often recount a global lack of control in their lives (Liu et al., 2016). Deficits related to affect and motivation could play an important role for this loss of control. Indeed, a core feature of major depressive disorder is anhedonia, which can be described as reduced motivation and ability to experience pleasure (Rizvi et al., 2016). This symptom has been linked to reduced reward sensitivity, reduced dopamine transmission, and structural and functional abnormalities including reduced gray matter volume and diminished reward signals in the striatum (Kumar et al., 2008; Wacker et al., 2009; Pizzagalli et al., 2010; Treadway and Zald, 2011). It has been proposed that the perceived control deficits in individuals with depression might reflect a more realistic view of events, so-called depressive realism (Alloy and Abramson, 1979; Alloy et al., 1984). Whereas individuals without depression demonstrate an illusion of control, individuals with depression are more accurate in their judgment about controllability. However, this view has not been supported by more recent work in clinically depressed (Dobson and Pusch, 1995; Moore and Fresco, 2012; Venkatesh et al., 2018). Importantly, it has been found that depression is associated with poorer learning of contingencies (Chase et al., 2010), and structural abnormalities in corticostriatal regions relevant to instrumental contingency learning (Baumann et al., 1999; Coryell et al., 2005; Drevets et al., 2008). Thus, impairments in instrumental contingency learning could provide an explanation for reduced perceived control in depression. However, a recent study also goes beyond instrumental contingency learning and suggests that that an impairment in the intrinsic value of choice may play a role in depression (Romaniuk et al., 2018). Specifically, the study demonstrates an association between subclinical depressive symptoms and reduced striatal anticipatory response to choice opportunity.

Reduced perceived control is considered a transdiagnostic feature across anxiety disorders (Gallagher et al., 2014a). In line with these findings, structural abnormalities in the ventromedial PFC have often been demonstrated in anxiety disorders (Kühn et al., 2011). Furthermore, reduced reward processing, as indicated by reduced signaling in the NAcc and vmPFC during a reward-based decision-making task, has been shown in patients with posttraumatic stress disorder (Sailer et al., 2008). Interestingly, some studies suggest increased striatal activation in relation to anxiety-related psychopathology, as well as increased striatal volume (Kühn et al., 2011; Ly et al., 2013). One explanation for this discrepancy is that reductions in reward processing could be a result of prolonged stress and behavioral dysfunctions leading to reductions in perceived control similar to that observed in depression; whereas increased striatal activation and volume could reflect an increased desire for control in aversive or ambiguous contexts. For instance, increased striatal activation and volume in relation to anxiety could be associated with increased vigilance for threat, or an intolerance for uncertainty (Ly et al., 2013; Kim et al., 2017). One clinical feature that is more uniquely related to anxiety is the fear of losing control. An intolerance for uncertainty and disrupted contingency learning in a dynamic environment in relation to anxiety (Browning et al., 2015; Kim et al., 2017; Piray et al., 2018), might together explain this fear of losing control as well as the subsequent maladaptive forms of control-seeking behavior, such as avoidance and compulsive behavior.

### Disorders Characterized by Increased Perceived Control

While reduced perceived control can have detrimental effects on behavior and wellbeing, the other extreme—an abnormal increase in perception of control—can be just as problematic. An illusion of control involving abnormalities in corticostriatal circuits and dopamine have been suggested to play a role in pathological gambling (Clark et al., 2013; Orgaz et al., 2013). Features, such as choice opportunity, instrumental action (e.g., opportunity to throw a roulette ball by yourself), and near-miss outcomes (e.g., close to winning the jackpot without actual success) are often used in the game to promote an illusion of control in gamblers. In the long run, this illusion could contribute to ‘loss chasing’ in pathological gambling, where individuals continue gambling to recover previous losses (Clark et al., 2013). Cognitive distortions typical in gambling have been associated with recruitment of the reward circuitry (Campbell-Meiklejohn et al., 2008; Clark et al., 2009; Xue et al., 2011). It has been found that individuals with pathological gambling tend to show increased illusion of control in associative learning task where the probability of a desired outcome is pseudorandomly determined independent from the actions taken (Orgaz et al., 2013). Evidence for altered reward processing in pathological gamblers have been inconsistent: both blunted as well as increased neural responses to monetary and nonmonetary rewards have been found to be associated with this pathological gambling (Reuter et al., 2005; Balodis et al., 2012; van Holst et al., 2012). However, when neural responses to monetary versus nonmonetary stimuli (e.g., appetitive images) were compared, an increase in striatal signals was found to be associated with pathological gambling (Sescousse et al., 2013). This finding might suggest that a relative, increase in reward processing for monetary versus nonmonetary appetitive stimuli is more characteristic for pathological gambling rather than an increase in reward processing for monetary stimuli in absolute terms, although further work is necessary to build upon this idea.

Another clinical aspect related to illusion of control, is mania. Manic, as opposed to depressive phases of bipolar disorder are characterized by increased perceived control, as well as elevated mood, hyperactivity, and increased interest in goal-oriented behavior. These symptoms all fit well with the dopamine hypothesis, which has long been proposed as the theory to understand bipolar disorder (Ashok et al., 2017). According to this hypothesis, the opposing poles of this disorder could potentially be explained by opposite alterations in dopaminergic function: while hypodopaminergia could underlie depression, hyperdopaminergia might underlie mania. A failure of dopamine receptor and transporter homeostasis have been suggested to underlie bipolar disorder (Ashok et al., 2017). Additionally, hyperactivation in corticostriatal circuits during cue-induced reward anticipation has been demonstrated in manic patients (Bermpohl et al., 2009; Singh et al., 2013).

### Perceived Control as a Target for Treatment

Given that the loss or lack of perceived control is a core feature in many psychiatric disorders, focusing on boosting perceived control may be helpful for some disorders. Although there are preliminary indications that changes in perceived control is a working mechanism underlying cognitive behavioral therapies targeting anxiety disorders, what aspects of these therapies are critical to change the perception of control remain unclear (Gallagher et al., 2014b). The proposed reward-based framework may provide some leads to specifically target key reward-processing elements so as to restore perception of control. Stemming from this framework, it could be argued that providing choice, boosting instrumental goal-directed behavior, or promoting reinforcement could serve as promising ways to enhance the perception of control. For example, previous work targeting choice opportunity has shown some success in patient-controlled analgesia postoperative patients using choice provision (Ballantyne et al., 2018).

Furthermore, the reward-based framework could help us understand the mechanisms of actions underlying existing interventions. One of the most effective treatments for depression, behavioral activation therapy, (Jacobson et al., 1996; Dimidjian et al., 2006; Hopko et al., 2011), is based on structured attempts to increase overt behaviors that potentially bring patients into contact with reinforcing environmental contingencies (Hopko et al., 2003). The framework highlights the potential of this therapeutic procedure to influence perceived control via manipulations of instrumental contingency as well as simply increasing reinforcement. More research is needed to test the working mechanisms of behavioral activation therapy directly. These insights will not only help to improve the efficacy of the existing treatment protocols, but it could also inform us on the potential utility of behavioral activation therapy for other disorders characterized by perceived control deficits, such as anxiety disorders. Such knowledge could be relevant to the development of novel transdiagnostic treatments, which has shown to be a promising type of treatment innovation (Barlow et al., 2017).

Similarly, growth mindset interventions are focused on promoting the belief that an ability is improvable rather than fixed (Grant and Dweck, 2003). A central aspect behind a growth mindset is perceiving control via a belief in instrumental contingency. Rather than setting ability-linked goals, active learning goals can be formulated that put an explicit emphasis on learning, development, and seeking to master challenges. These goals enable the individual to see aversive outcomes as information to improve learning, rather than as indicators of stable low ability (Moser et al., 2011). Such interventions promote intrinsic motivation and perceived control (Grant and Dweck, 2003). Furthermore, growth mindsets have been demonstrated to have beneficial effects for coping with negative affect and reducing physiological stress responses to negative events (Yeager et al., 2016). These growth mindset interventions have been mainly used in education, but could potentially have beneficial effects in psychiatric disorders characterized by perceived controldeficits.

## Conclusion

Through the reward-based framework of perceived control, we have highlighted how choice opportunity, instrumental contingency, and reward rate could individually and collectively contribute to specific and general perceived control. In particular, we discussed how both animal and human research has shown the contribution of corticostriatal circuits and dopamine to these key elements of perceiving control. Apart from focusing on factors contributing to perceived control, we have also highlighted some work demonstrating that the alterations of these key elements could influence affect regulation and behavior. Insights into the consequences of perceived control are relevant, particularly given that its affective and behavioral consequences could, along with future decision making, enter into a vicious and pathological cycle.

Further research is needed to elucidate how interactions within the corticostriatal circuits play a role in perceived control. We hypothesize that choice opportunity may bias instrumental action-selection via input from the striatum, which is the main candidate to integrate motivational and affective value with instrumental actions. It remains to be tested how prefrontal and striatal regions involved in perceived control exactly interact. For these investigations, we would need to employ a multimodal approach by combining well-validated behavioral study procedures with neural interventions (e.g., brain stimulation) and sophisticated data-processing tools such as dynamic causal modeling to allow for inferences on the causal neural mechanisms (Wang et al., 2016). Furthermore, direct manipulations of the dopaminergic and serotonergic systems in pharmacological studies could provide more information on the role of these neuromodulatory systems in regulating the relationship between perceived control and adaptive behavior. Another interesting open question is how general perceived control and its subjective value in individuals is coded in the brain and how it may change in context-dependent perceived control.

Finally, understanding perceived control has clinical implications. Given that abnormalities in these reward-related processes have often been observed in many psychopathological states, such research could help us better understand the role of perceived control in their etiology and maintenance. Investigating how perceived control is impaired across a range of psychiatric disorders, could lead to more insights into phenotypes and how individual differences in key reward-related elements might serve as a predictor for susceptibility to these disorders.

## Author Contributions

VL took the lead in writing the manuscript. All authors conceived of the presented idea, provided critical feedback, and helped to shape the manuscript.

## Conflict of Interest Statement

The authors declare that the research was conducted in the absence of any commercial or financial relationships that could be construed as a potential conflict of interest.

## References

[B1] Abramson L. Y. Seligman M. E. P. Teasdale J. D. (1978). Learned helplessness in humans: critique and reformulation. *J. Abnorm. Psychol.* 87 49–74. 10.1037/0021-843X.87.1.49 649856

[B2] Alloy L. B. Abramson L. Y. (1979). Judgment of contingency in depressed and nondepressed students: sadder but wiser? *J. Exp. Psychol. Gen.* 108 441–485. 10.1037/0096-3445.108.4.441 528910

[B3] Alloy L. B. Peterson C. Abramson L. Y. Seligman M. E. (1984). Attributional style and the generality of learned helplessness. *J. Pers. Soc. Psychol.* 46 681–687. 10.1037/0022-3514.46.3.681 6707869

[B4] AmatJ.BarattaM. V.PaulE.BlandS. T.WatkinsL. R.MaierS. F. (2005). Medial prefrontal cortex determines how stressor controllability affects behavior and dorsal raphe nucleus. *Nat. Neurosci.* 8 365–371. 10.1038/nn1399 15696163

[B5] Amat J. Christianson J. P. Aleksejev R. M. Kim J. Richeson K. R. Watkins L. R. (2014). Control over a stressor involves the posterior dorsal striatum and the act/outcome circuit. *Eur. J. Neurosci.* 40 2352–2358. 10.1111/ejn.12609 24862585 PMC4804456

[B6] Amat J. Paul E. Zarza C. Watkins L. R. Maier S. F. (2006). Previous experience with behavioral control over stress blocks the behavioral and dorsal raphe nucleus activating effects of later uncontrollable stress: role of the ventral medial prefrontal cortex. *J. Neurosci.* 26 13264–13272. 10.1523/JNEUROSCI.3630-06.2006 17182776 PMC6675012

[B7] Ashok A. H. Marques T. R. Jauhar S. Nour M. M. Goodwin G. M. Young A. H. (2017). The dopamine hypothesis of bipolar affective disorder: the state of the art and implications for treatment. *Mol. Psychiatry* 22 666–679. 10.1038/mp.2017.16 28289283 PMC5401767

[B8] Averill J. R. (1973). Personal control over aversive stimuli and its relationship to stress. *Psychol. Bull.* 80 286–303. 10.1037/h0034845

[B9] Ballantyne J. C. Carr D. B. Chalmers T. C. Dear K. B. G. Angelillo I. F. Mosteller F. (2018). Postoperative patient-controlled analgesia: meta-analyses of initial randomized control trials. *J. Clin. Anesth.* 5 182–193. 10.1016/0952-8180(93)90013-5 8318237

[B10] Balleine B. W. Dickinson A. (1998). Goal-directed instrumental action: contingency and incentive learning and their cortical substrates. *Neuropharmacology* 37 407–419. 10.1016/S0028-3908(98)00033-1 9704982

[B11] Balodis I. M. Kober H. Worhunsky P. D. Stevens M. C. Pearlson G. D. Potenza M. N. (2012). Diminished fronto-striatal activity during processing of monetary rewards and losses in pathological gambling. *Biol. Psychiatry* 71 749–757. 10.1016/j.biopsych.2012.01.006 22336565 PMC3460522

[B12] Bandura A. (1977). Self-efficacy: toward a unified theory of behavioral change. *Psychol. Rev.* 84 191–215. 847061 10.1037//0033-295x.84.2.191

[B13] Barlow D. H. Farchione T. J. Bullis J. R. Gallagher M. W. Murray-Latin H. Sauer-Zavala S. (2017). The unified protocol for transdiagnostic treatment of emotional disorders compared with diagnosis-specific protocols for anxiety disorders: a randomized clinical trial. *JAMA Psychiatry* 74 875–884. 10.1001/jamapsychiatry.2017.2164 28768327 PMC5710228

[B14] Baumann B. Danos P. Krell D. Diekmann S. Leschinger A. Stauch R. (1999). Reduced volume of limbic system-affiliated basal ganglia in mood disorders: preliminary data from a postmortem study. *J. Neuropsychiatry Clin. Neurosci.* 11 71–78. 10.1176/jnp.11.1.71 9990559

[B15] Beierholm U. Guitart-masip M. Economides M. Chowdhury R. Dolan R. Dayan P. (2013). Dopamine modulates reward-related vigor. *Neuropsychopharmacology* 38 1495–1503. 10.1038/npp.2013.48 23419875 PMC3682144

[B16] Bermpohl F. Kahnt T. Dalanay U. Hägele C. Sajonz B. Wegner T. (2009). Altered representation of expected value in the orbitofrontal cortex in mania. *Hum. Brain Mapp.* 31 958–969. 10.1002/hbm.20909 19950195 PMC6870977

[B17] Berridge K. C. (2007). The debate over dopamine’s role in reward: the case for incentive salience. *Psychopharmacology* 191 391–431. 10.1007/s00213-006-0578-x 17072591

[B18] Boeke E. A. Moscarello J. M. Ledoux X. J. E. Phelps E. A. Hartley C. A. (2017). Active avoidance: neural mechanisms and attenuation of pavlovian conditioned responding. *J. Neurosci.* 37 4808–4818. 10.1523/JNEUROSCI.3261-16.2017 28408411 PMC5426570

[B19] Boureau Y.-L. Dayan P. (2011). Opponency revisited: competition and cooperation between dopamine and serotonin. *Neuropsychopharmacology* 36 74–97. 10.1038/npp.2010.151 20881948 PMC3055522

[B20] Bown N. J. Read D. Summers B. (2003). The lure of choice. *J. Behav. Decis. Mak.* 16 297–308. 10.1002/bdm.447

[B21] Bromberg-Martin E. S. Matsumoto M. Hikosaka O. (2010). Distinct tonic and phasic anticipatory activity in lateral habenula and dopamine neurons. *Neuron* 67 144–155. 10.1016/j.neuron.2010.06.016 20624598 PMC2905384

[B22] Browning M. Behrens T. E. Jocham G. O’Reilly J. X. Bishop S. J. (2015). Anxious individuals have difficulty learning the causal statistics of aversive environments. *Nat. Neurosci.* 18 590–596. 10.1038/nn.3961 25730669 PMC4644067

[B23] Cabib S. Puglisi-Allegra S. (2012). The mesoaccumbens dopamine in coping with stress. *Neurosci. Biobehav. Rev.* 36 79–89. 10.1016/j.neubiorev.2011.04.012 21565217

[B24] Campbell-Meiklejohn D. K. Woolrich M. W. Passingham R. E. Rogers R. D. (2008). Knowing when to stop: the brain mechanisms of chasing losses. *Biol. Psychiatry* 63 293–300. 10.1016/j.biopsych.2007.05.014 17662257

[B25] Catania A. C. Sagvolden T. (1980). Preference for free choice over forced choice in pigeons. *J. Exp. Anal. Behav.* 34 77–86. 10.1901/jeab.1980.34-77 16812181 PMC1332946

[B26] Chase H. W. Frank M. J. Michael A. Bullmore E. T. Sahakian B. J. Robbins T. W. (2010). Approach and avoidance learning in patients with major depression and healthy controls: relation to anhedonia. *Psychol. Med.* 40 433–440. 10.1017/S0033291709990468 19607754

[B27] Chorpita B. F. Barlow D. H. (1998). The development of anxiety: the role of control in the early environment. *Psychol. Bull.* 124 3–21. 10.1037/0033-2909.124.1.3 9670819

[B28] Christianson J. P. Greenwood B. N. (2014). Stress-protective neural circuits: not all roads lead through the prefrontal cortex. *Stress* 17 1–12. 10.3109/10253890.2013.794450 23574145

[B29] Clark L. Averbeck B. Payer D. Sescousse G. Winstanley C. A. Xue G. (2013). Pathological choice: the neuroscience of gambling and gambling addiction. *J. Neurosci.* 33 17617–17623. 10.1523/JNEUROSCI.3231-13.2013 24198353 PMC3858640

[B30] Clark L. Lawrence A. J. Astley-Jones F. Gray N. (2009). Gambling near-misses enhance motivation to gamble and recruit win-related brain circuitry. *Neuron* 61 481–490. 10.1016/j.neuron.2008.12.031 19217383 PMC2658737

[B31] Cockburn J. Collins A. G. Frank M. J. (2014). A reinforcement learning mechanism responsible for the valuation of free choice. *Neuron* 83 551–557. 10.1016/j.neuron.2014.06.035 25066083 PMC4126879

[B32] Cools R. Nakamura K. Daw N. D. (2011). Serotonin and dopamine: unifying affective, activational, and decision functions. *Neuropsychopharmacology* 36 98–113. 10.1038/npp.2010.121 20736991 PMC3055512

[B33] Corbit L. H. Balleine B. W. (2003). The role of prelimbic cortex in instrumental conditioning. *Behav. Brain Res.* 146 145–157. 10.1016/j.bbr.2003.09.023 14643467

[B34] Corbit L. H. Balleine B. W. (2011). The general and outcome-specific forms of pavlovian-instrumental transfer are differentially mediated by the nucleus accumbens core and shell. *J. Neurosci.* 31 11786–11794. 10.1523/JNEUROSCI.2711-11.2011 21849539 PMC3208020

[B35] Coryell W. Nopoulos P. Drevets W. Wilson T. Andreasen N. C. (2005). Subgenual prefrontal cortex volumes in major depressive disorder and schizophrenia: diagnostic specificity and prognostic implications. *Am. J. Psychiatry* 162 1706–1712. 10.1176/appi.ajp.162.9.1706 16135631

[B36] Cuthbert B. N. Insel T. R. (2013). Toward the future of psychiatric diagnosis: the seven pillars of RDoC. *BMC Med.* 11:126. 10.1186/1741-7015-11-126 23672542 PMC3653747

[B37] De Brabander B. Declerck C. H. (2004). A possible role of central dopamine metabolism associated with individual differences in locus of control. *Pers. Individ. Dif.* 37 735–750. 10.1016/j.paid.2003.11.001

[B38] Deci E. Ryan R. M. (1985). *Intrinsic Motivation and Self-Determination in Human Behavior.* Berlin: Springer.

[B39] Declerck C. H. Boone C. De Brabander B. (2006). On feeling in control: a biological theory for individual differences in control perception. *Brain Cogn.* 62 143–176. 10.1016/j.bandc.2006.04.004 16806623

[B40] Delgado M. R. Jou R. L. Ledoux J. E. Phelps E. A. (2009). Avoiding negative outcomes: tracking the mechanisms of avoidance learning in humans during fear conditioning. *Front. Behav. Neurosci.* 3:33. 10.3389/neuro.08.033.2009 19847311 PMC2762377

[B41] Dickinson A. Balleine B. (1994). Motivational control of goal-directed action. *Anim. Learn. Behav.* 22 1–18. 10.3758/BF03199951

[B42] Dimidjian S. Hollon S. D. Dobson K. S. Schmaling K. B. Kohlenberg R. J. Addis M. E. (2006). Randomized trial of behavioral activation, cognitive therapy, and antidepressant medication in the acute treatment of adults with major depression. *J. Consult. Clin. Psychol.* 74 658–670. 10.1037/0022-006X.74.4.658 16881773

[B43] Dobson K. S. Pusch D. (1995). A test of the depressive realism hypothesis in clinically depressed subjects. *Cogn. Ther. Res.* 19 179–194. 10.1007/BF02229693 17362430

[B44] Drevets W. C. Price J. L. Furey M. L. (2008). Brain structural and functional abnormalities in mood disorders: implications for neurocircuitry models of depression. *Brain Struct. Funct.* 213 93–118. 10.1007/s00429-008-0189-x 18704495 PMC2522333

[B45] Eitam B. Kennedy P. M. Higgins E. T. (2013). Motivation from control. *Exp. Brain Res.* 229 475–484. 10.1007/s00221-012-3370-7 23288323

[B46] Elliot A. J. Dweck C. S. (2005). *Handbook of Competence and Motivation.* New York, NY: The Guilford Press.

[B47] Faure A. Reynolds S. M. Richard J. M. Berridge K. C. (2009). Mesolimbic dopamine in desire and dread: enabling motivation to be generated by localized glutamate disruptions in nucleus accumbens. *J. Nuerosci.* 28 7184–7192. 10.1523/JNEUROSCI.4961-07.2008 PMC2519054 18614688

[B48] Floresco S. B. West A. R. Ash B. Moore H. Grace A. A. (2003). Afferent modulation of dopamine neuron firing differentially regulates tonic and phasic dopamine transmission. *Nat. Neurosci.* 6 968–973. 10.1038/nn1103 12897785

[B49] Fujiwara J. Usui N. Park S. Q. Williams T. Iijima T. Taira M. (2013). Value of freedom to choose encoded by the human brain. *J. Neurophysiol.* 110 1915–1929. 10.1152/jn.01057.2012 23864380 PMC3798941

[B50] Gallagher M. W. Bentley K. H. Barlow D. H. (2014a). Perceived control and vulnerability to anxiety disorders: a meta-analytic review. *Cogn. Ther. Res.* 38 571–584. 10.1007/s10608-014-9624-x

[B51] Gallagher M. W. Naragon-gainey K. Brown T. A. (2014b). Perceived control is a transdiagnostic predictor of cognitive – behavior therapy outcome for anxiety disorders. *Cogn. Ther. Res.* 8 10–22. 10.1007/s10608-013-9587-3 24563563 PMC3927880

[B52] Goodkin F. (1976). Rats learn the relationship between responding and environmental events: an expansion of the learned helplessness hypothesis. *Learn. Motiv.* 7 382–393. 10.1016/0023-9690(76)90044-8

[B53] Grant H. Dweck C. S. (2003). Clarifying achievement goals and their impact. *J. Pers. Soc. Psychol.* 85 541–553. 10.1037/0022-3514.85.3.541 14498789

[B54] Griffiths K. R. Morris R. W. Balleine B. W. (2014). Translational studies of goal-directed action as a framework for classifying deficits across psychiatric disorders. *Front. Syst. Neurosci.* 8:101. 10.3389/fnsys.2014.00101 24904322 PMC4033402

[B55] Hartley C. A. Gorun A. Reddan M. C. Ramirez F. Phelps E. A. (2014). Stressor controllability modulates fear extinction in humans. *Neurobiol. Learn. Mem.* 113 149–156. 10.1016/j.nlm.2013.12.003 24333646 PMC4053478

[B56] Hollerman J. R. Schultz W. (1998). Dopamine neurons report an error in the temporal prediction of reward during learning. *Nat. Neurosci.* 1 304–309. 10.1038/1124 10195164

[B57] Hopko D. R. Armento M. E. A. Robertson S. M. C. Ryba M. M. Carvalho J. P. Colman L. K. (2011). Brief behavioral activation and problem-solving therapy for depressed breast cancer patients: randomized trial. *J. Consult. Clin. Psychol.* 79 834–849. 10.1037/a0025450 21988544

[B58] Hopko D. R. Lejuez C. W. Ruggiero K. J. Eifert G. H. (2003). Contemporary behavioral activation treatments for depression: procedures, principles, and progress. *Clin. Psychol. Rev.* 23 699–717. 10.1016/S0272-7358(03)00070-9 12971906

[B59] Huys Q. J. M. Cools R. Golzer M. Friedel E. Heinz A. Dolan R. J. (2011). Disentangling the roles of approach, activation and valence in instrumental and Pavlovian responding. *PLoS Comput. Biol.* 7:e1002028. 10.1371/journal.pcbi.1002028 21556131 PMC3080848

[B60] Huys Q. J. M. Daw N. D. Dayan P. (2015). Depression: a decision-theoretic analysis. *Annu. Rev. Neurosci.* 38 1–23. 10.1146/annurev-neuro-071714-033928 25705929

[B61] Huys Q. J. M. Dayan P. (2009). A Bayesian formulation of behavioral control. *Cognition* 113 314–328. 10.1016/j.cognition.2009.01.008 19285311

[B62] Jacobson N. S. Dobson K. S. Truax P. A. Addis M. E. Koerner K. Gollan J. K. (1996). A component analysis of cognitive-behavioral treatment for depression. *J. Consult. Clin. Psychol.* 79 834–849. 10.1037/0022-006X.64.2.295 8871414

[B63] Joiner T. E. Wingate L. R. Otamendi A. (2005). An interpersonal addendum to the hopelessness theory of depression: hopelessness as a stress and depression generator. *J. Soc. Clin. Psychol.* 24 649–664. 10.1521/jscp.2005.24.5.649

[B64] Kayser A. S. Mitchell J. M. Weinstein D. Frank M. J. (2014). Dopamine, locus of control, and the exploration-exploitation tradeoff. *Neuropsychopharmacology* 40 454–462. 10.1038/npp.2014.193 25074639 PMC4443960

[B65] Kerr D. L. Mclaren D. G. Mathy R. M. Nitschke J. B. (2012). Controllability modulates the anticipatory response in the human ventromedial prefrontal cortex. *Front. Psychol.* 3:557. 10.3389/fpsyg.2012.00557 23550176 PMC3582324

[B66] Kim M. J. Shin J. Taylor J. M. Mattek A. M. Chavez S. J. Whalen P. J. (2017). Intolerance of uncertainty predicts increased striatal volume. *Emotion* 17 895–899. 10.1037/emo0000331 28517947 PMC5573619

[B67] Kühn S. Haggard P. Brass M. (2014). Differences between endogenous and exogenous emotion inhibition in the human brain. *Brain Struct. Funct.* 219 1129–1138. 10.1007/s00429-013-0556-0 23644585

[B68] Kühn S. Schubert F. Gallinat J. (2011). Structural correlates of trait anxiety: reduced thickness in medial orbitofrontal cortex accompanied by volume increase in nucleus accumbens. *J. Affect. Disord.* 134 315–319. 10.1016/j.jad.2011.06.003 21705088

[B69] Kumar P. Waiter G. Ahearn T. Milders M. Reid I. Steele J. D. (2008). Abnormal temporal difference reward-learning signals in major depression. *Brain* 131 2084–2093. 10.1093/brain/awn136 18579575

[B70] Kurniawan I. T. Guitart-Masip M. Dolan R. J. (2011). Dopamine and effort-based decision making. *Front. Neurosci.* 5:81 10.3389/fnins.2011.00081 PMC3122071 21734862

[B71] Leotti L. A. Delgado M. R. (2011). The inherent reward of choice. *Psychol. Sci.* 22 1310–1318. 10.1177/0956797611417005 21931157 PMC3391581

[B72] Leotti L. A. Delgado M. R. (2014). The value of exercising control over monetary gains and losses. *Psychol. Sci.* 25 596–604. 10.1177/0956797613514589 24390827 PMC3970926

[B73] Leotti L. A. Iyengar S. S. Ochsner K. N. (2010). Born to choose: the origins and value of the need for control. *Trends Cogn. Sci.* 14 457–463. 10.1016/j.tics.2010.08.001 20817592 PMC2944661

[B74] Lieder F. Goodman N. D. Huys Q. J. M. (2013). “Learned helplessness and generalization,” in *Proceedings of the 35th Annual Conference of the Cognitive Science Society*, Austin, TX, 900–905.

[B75] Liljeholm M. Molloy C. J. O’Doherty J. P. (2012). Dissociable brain systems mediate vicarious learning of stimulus–response and action–outcome contingencies. *J. Neurosci.* 32 9878–9886. 10.1523/JNEUROSCI.0548-12.2012 22815503 PMC3428877

[B76] Liljeholm M. Tricomi E. O’Doherty J. P. Balleine B. W. (2011). Neural correlates of instrumental contingency learning: differential effects of action-reward conjunction and disjunction. *J. Neurosci.* 31 2474–2480. 10.1523/JNEUROSCI.3354-10.2011 21325514 PMC3269757

[B77] Liu R. T. Kleiman E. M. Nestor B. A. Cheek S. M. (2016). The hopelessness theory of depression: a quarter century in review. *Clin. Psychol.* 22 345–365. 10.1111/cpsp.12125 26709338 PMC4689589

[B78] Lohani S. Martig A. K. Underhill S. M. DeFrancesco A. Roberts M. J. Rinaman L. (2018). Burst activation of dopamine neurons produces prolonged post-burst availability of actively released dopamine. *Neuropsychopharmacology* 43 2083–2092. 10.1038/s41386-018-0088-7 29795245 PMC6098082

[B79] Ly V. Cools R. Roelofs K. (2013). Aversive disinhibition of behavior and striatal signaling in social avoidance. *Soc. Cogn. Affect. Neurosci.* 9 1530–1536. 10.1093/scan/nst145 23986267 PMC4187270

[B80] Maier S. F. Seligman M. E. P. (2016). Learned helplessness at fifty: insights from neuroscience. *Psychol. Rev.* 123 349–367. 10.1037/rev0000033 27337390 PMC4920136

[B81] Maier S. F. Watkins L. R. (2005). Stressor controllability and learned helplessness: the roles of the dorsal raphe nucleus, serotonin, and corticotropin-releasing factor. *Neurosci. Biobehav. Rev.* 29 829–841. 10.1016/j.neubiorev.2005.03.021 15893820

[B82] Matute H. (1996). Illusion of control: detecting response-outcome independence in analytic but not in naturalistic conditions. *Psychol. Sci.* 7 289–293. 10.1111/j.1467-9280.1996.tb00376.x

[B83] Miller W. R. Seligman M. E. P. (1975). Depression and learned helplessness in man. *J. Abnorm. Psychol.* 84 228–238. 10.1037/h0076720 1169264

[B84] Mineka S. Gunnar M. Champoux M. (1986). Control and early socioemotional development: infant rhesus monkeys reared in controllable versus uncontrollable environments. *Child Dev.* 57 1241–1256.

[B85] Mineka S. Hendersen R. W. (1985). Controllability and predictability in acquired motivation. *Annu. Rev. Psychol.* 36 495–529. 10.1146/annurev.ps.36.020185.002431 3919637

[B86] Mistry P. Liljeholm M. (2016). Instrumental divergence and the value of control. *Sci. Rep.* 6:36295. 10.1038/srep36295 27811969 PMC5095609

[B87] Moore M. T. Fresco D. M. (2012). Depressive realism: a meta-analytic review. *Clin. Psychol. Rev.* 32 496–509. 10.1016/j.cpr.2012.05.004 22717337

[B88] Moscarello J. M. Hartley C. A. (2017). Agency and the calibration of motivated behavior. *Trends Cogn. Sci.* 21 725–735. 10.1016/j.tics.2017.06.008 28693961

[B89] Moser J. S. Schroder H. S. Heeter C. Moran T. P. Lee Y.-H. (2011). Mind your errors: evidence for a neural mechanism linking growth mind-set to adaptive posterror adjustments. *Psychol. Sci.* 22 1484–1489. 10.1177/0956797611419520 22042726

[B90] Murayama K. Matsumoto M. Izuma K. Sugiura A. Ryan R. M. Deci E. L. (2013). How self-determined choice facilitates performance: a key role of the ventromedial prefrontal cortex. *Cereb. Cortex* 25 1241–1251. 10.1093/cercor/bht317 24297329

[B91] Murty V. P. DuBrow S. Davachi L. (2015). The simple act of choosing influences declarative memory. *J. Neurosci.* 35 6255–6264. 10.1523/JNEUROSCI.4181-14.2015 25904779 PMC4405547

[B92] Niv Y. Daw N. D. Joel D. Dayan P. (2007). Tonic dopamine: opportunity costs and the control of response vigor. *Psychopharmacology* 191 507–520. 10.1007/s00213-006-0502-4 17031711

[B93] Orgaz C. Estévez A. Matute H. (2013). Pathological gamblers are more vulnerable to the illusion of control in a standard associative learning task. *Front. Psychol.* 4:306. 10.3389/fpsyg.2013.00306 23785340 PMC3683617

[B94] Panksepp J. (1998). *Affective Neuroscience: The Foundations of Human and Animal Emotions.* New York, NY: Oxford University Press.

[B95] Phillips P. E. M. Walton M. E. Jhou T. C. (2007). Calculating utility: preclinical evidence for cost–benefit analysis by mesolimbic dopamine. *Psychopharmacology* 191 483–495. 10.1007/s00213-006-0626-6 17119929

[B96] Phillips P. E. M. Wightman R. M. (2004). Extrasynaptic dopamine and phasic neuronal activity. *Nat. Neurosci.* 7:199. 10.1038/nn0304-199a 14983175

[B97] Piray P. Ly V. Roelofs K. Cools R. Toni I. (2018). Emotionally aversive cues suppress neural systems underlying optimal learning in socially anxious individuals. *J. Neurosci.* 10.1523/JNEUROSCI.1394-18.2018 [Epub ahead of print]. 30559152 PMC6381249

[B98] Pizzagalli D. A. Holmes A. J. Dillon D. G. Goetz E. L. Birk J. L. Bogdan R. (2010). Reduced caudate and nucleus accumbens response to rewards in unmedicated individuals with major depressive disorder. *Am. J. Psychiatry* 166 702–710. 10.1176/appi.ajp.2008.08081201 19411368 PMC2735451

[B99] Reuter J. Raedler T. Rose M. Hand I. Gläscher J. Büchel C. (2005). Pathological gambling is linked to reduced activation of the mesolimbic reward system. *Nat. Neurosci.* 8 147–148. 10.1038/nn1378 15643429

[B100] Rigoli F. Pezzulo G. Dolan R. J. (2016). Prospective and Pavlovian mechanisms in aversive behaviour. *Cognition* 146 415–425. 10.1016/j.cognition.2015.10.017 26539969 PMC4675632

[B101] Rizvi S. J. Pizzagalli D. A. Sproule B. A. Kennedy S. H. (2016). Assessing anhedonia in depression: potentials and pitfalls. *Neurosci. Biobehav. Rev.* 65 21-35. 10.1016/j.neubiorev.2016.03.004 26959336 PMC4856554

[B102] Romaniuk L. Sandu A.-L. Waiter G. D. McNeil C. J. Shen X. Harris M. A. (2018). The neurobiology of personal control during reward learning and its relationship to mood. *Biol. Psychiatry Cogn. Neurosci. Neuroimaging* 10.1016/j.bpsc.2018.09.015 [Epub ahead of print]. 30470583 PMC6374985

[B103] Rotter J. B. (1966). Generalized expectancies for internal versus external control of reinforcement. *Psychol. Monogr. Gen. Appl.* 80 1–28. 10.1037/h0092976 5340840

[B104] Sailer U. Robinson S. Ph F. Fischmeister S. Dorothea K. Oppenauer C. (2008). Altered reward processing in the nucleus accumbens and mesial prefrontal cortex of patients with posttraumatic stress disorder. *Neuropsychologia* 46 2836–2844. 10.1016/j.neuropsychologia.2008.05.022 18597797

[B105] Salamone J. D. (2018). Dopamine, effort-based choice, and behavioral economics: basic and translational research. *Front. Behav. Neurosci.* 12:52. 10.3389/fnbeh.2018.00052 29628879 PMC5876251

[B106] Salomons T. V. Johnstone T. Backonja M.-M. Davidson R. J. (2004). Perceived controllability modulates the neural response to pain. *J. Neurosci.* 24 7199–7203. 10.1523/JNEUROSCI.1315-04.2004 15306654 PMC6729173

[B107] Schultz W. Dayan P. Montague P. R. (1997). A neural substrate of prediction and reward. *Science* 275 1593–1599. 10.1126/science.275.5306.1593 9054347

[B108] Sescousse G. Barbalat G. Domenech P. Dreher J.-C. (2013). Imbalance in the sensitivity to different types of rewards in pathological gambling. *Brain* 136 2527–2538. 10.1093/brain/awt126 23757765

[B109] Shanks D. R. Dickinson A. (1991). Instrumental judgment and performance under variations in action-outcome contingency and contiguity. *Mem. Cogn.* 19 353–360. 10.3758/BF03197139 1895945

[B110] Singh M. K. Chang K. D. Kelley R. G. Cui X. Sherdell L. Howe M. E. (2013). Reward processing in adolescents with bipolar I disorder. *J. Am. Acad. Child Adolesc. Psychiatry* 52 68–83. 10.1016/j.jaac.2012.10.004 23265635 PMC3530159

[B111] Skinner E. (2007). “Perceived control: engagement, coping, and development,” in *21st Century Education: A Reference Handbook*, ed. Good T. L. (Newbury Park, CA: Sage Publications).

[B112] Skinner E. A. Connell J. Deci E. Dweck C. Heckhausen J. Ryan R. (1996). A guide to constructs of control. *J. Pers. Soc. Psychol.* 71 549–570. 10.1037/0022-3514.71.3.549 8831161

[B113] Stillman T. F. Baumeister R. F. Vohs K. D. Lambert N. M. Fincham F. D. Brewer L. E. (2010). Personal philosophy and personnel achievement: belief in free will predicts better job performance. *Soc. Psychol. Pers. Sci.* 1 43–50. 10.1177/1948550609351600

[B114] Suzuki S. (1997). Effects of number of alternatives on choice in humans. *Behav. Process.* 39 205–214. 10.1016/S0376-6357(96)00049-6 24896966

[B115] Suzuki S. (1999). Selection of forced- and free-choice by monkeys (*Macaca fascicularis*). *Percept. Mot. Skills* 88 242–250. 10.2466/pms.1999.88.1.242

[B116] Swart J. C. Frobose M. I. Cook J. L. Geurts D. E. M. Frank M. J. Cools R. (2017). Catecholaminergic challenge uncovers distinct Pavlovian and instrumental mechanisms of motivated (in)action. *eLife* 6:e22169. 10.7554/eLife.22169.001 28504638 PMC5432212

[B117] Tanaka S. C. Balleine B. W. O’Doherty J. P. (2008). Calculating consequences: brain systems that encode the causal effects of actions. *J. Neurosci.* 28 6750–6755. 10.1523/JNEUROSCI.1808-08.2008 18579749 PMC3071565

[B118] Teodorescu K. Erev I. (2014). Learned helplessness and learned prevalence: exploring the causal relations among perceived controllability, reward prevalence, and exploration. *Psychol. Sci.* 25 1861–1869. 10.1177/0956797614543022 25193942

[B119] Thorndike E. L. (1911). *Animal Intelligence; Experimental Studies.* New York, NY: The Macmillan company 10.5962/bhl.title.55072

[B120] Tobias-webb J. Limbrick-oldfield E. H. Gillan C. M. James W. Aitken M. R. F. Clark L. (2017). Let me take the wheel: illusory control and sense of agency. *Q. J. Exp. Psychol.* 70 1732–1746. 10.1080/17470218.2016.1206128 27376771 PMC5399809

[B121] Tobler P. N. Fiorillo C. D. Schultz W. (2005). Adaptive coding of reward value by dopamine neurons. *Science* 307 1642–1645. 10.1126/science.1105370 15761155

[B122] Treadway M. T. Zald D. H. (2011). Reconsidering anhedonia in depression: lessons from translational neuroscience. *Neurosci. Biobehav. Rev.* 35 537–555. 10.1016/j.neubiorev.2010.06.006 20603146 PMC3005986

[B123] Valentin V. V. Dickinson A. O’Doherty J. P. (2007). Determining the neural substrates of goal-directed learning in the human brain. *J. Neurosci.* 27 4019–4026. 10.1523/JNEUROSCI.0564-07.2007 17428979 PMC6672546

[B124] van Holst R. J. Veltman D. J. Büchel C. van den Brink W. Goudriaan A. E. (2012). Distorted expectancy coding in problem gambling: is the addictive in the anticipation? *Biol. Psychiatry* 71 741–748. 10.1016/j.biopsych.2011.12.030 22342105

[B125] Varela J. A. Wang J. Christianson J. P. Maier S. F. Cooper D. C. (2012). Control over stress, but not stress per se increases prefrontal cortical pyramidal neuron excitability. *J. Neurosci.* 32 12848–12853. 10.1523/JNEUROSCI.2669-12.2012 22973008 PMC3732060

[B126] Venkatesh S. Moulds M. L. Mitchell C. J. (2018). Testing for depressive realism in a clinically depressed sample. *Behav. Change* 35 108–122. 10.1017/bec.2018.12

[B127] Vertes R. P. (2004). Differential projections of the infralimbic and prelimbic cortex in the rat. *Synapse* 51 32–58. 10.1002/syn.10279 14579424

[B128] Wacker J. Dillon D. G. Pizzagalli D. A. (2009). The role of the nucleus accumbens and rostral anterior cingulate cortex in anhedonia: integration of resting EEG, fMRI, and volumetric techniques. *Neuroimage* 46 327–337. 10.1016/j.neuroimage.2009.01.058 19457367 PMC2686061

[B129] Wang K. S. Smith D. V. Delgado M. R. (2016). Using fMRI to study reward processing in humans: past, present, and future. *J. Neurophysiol.* 115 1664–1678. 10.1152/jn.00333.2015 26740530 PMC4808130

[B130] White R. W. (1959). Motivation reconsidered: the concept of competence. *Psychol. Rev.* 66 297–333. 10.1037/h0040934 13844397

[B131] Xue G. Lu Z. Levin I. P. Bechara A. (2011). An fMRI study of risk-taking following wins and losses: implications for the gambler’s fallacy. *Hum. Brain Mapp.* 32 271–281. 10.1002/hbm.21015 21229615 PMC3429350

[B132] Yeager D. S. Lee H. Y. Jamieson J. P. (2016). How to improve adolescent stress responses: insights from an integration of implicit theories and biopsychosocial models. *Psychol. Sci.* 27 1078–1091. 10.1177/0956797616649604 27324267 PMC4976003

[B133] Yin H. H. Knowlton B. J. Balleine B. W. (2005). Blockade of NMDA receptors in the dorsomedial striatum prevents action–outcome learning in instrumental conditioning. *Eur. J. Neurosci.* 22 505–512. 10.1111/j.1460-9568.2005.04219.x 16045503

